# Supplementary material for: Buffering of transcription rate by mRNA half-life is a conserved feature of Rett syndrome models
Source: Nat Commun. 2023 Apr 5;14:1896. doi: 10.1038/s41467-023-37339-6 (PMC10076348; doi:10.1038/s41467-023-37339-6)
Supplement: Supplementary file 1 — Supplmentary Information [file 41467_2023_37339_MOESM1_ESM.pdf]

Supplementary Information for:

Buffering of transcription rate by mRNA half-life is a conserved feature of Rett syndrome models.

Deivid C. Rodrigues[1], Marat Mufteev[1,2], Kyoko E. Yuki[3], Ashrut Narula[2,4], Wei Wei[1], Alina Piekna[1], Jiajie Liu[1], Peter Pasceri[1], Olivia S. Rissland[4,5], Michael D. Wilson[2,3], and James Ellis[1,2]

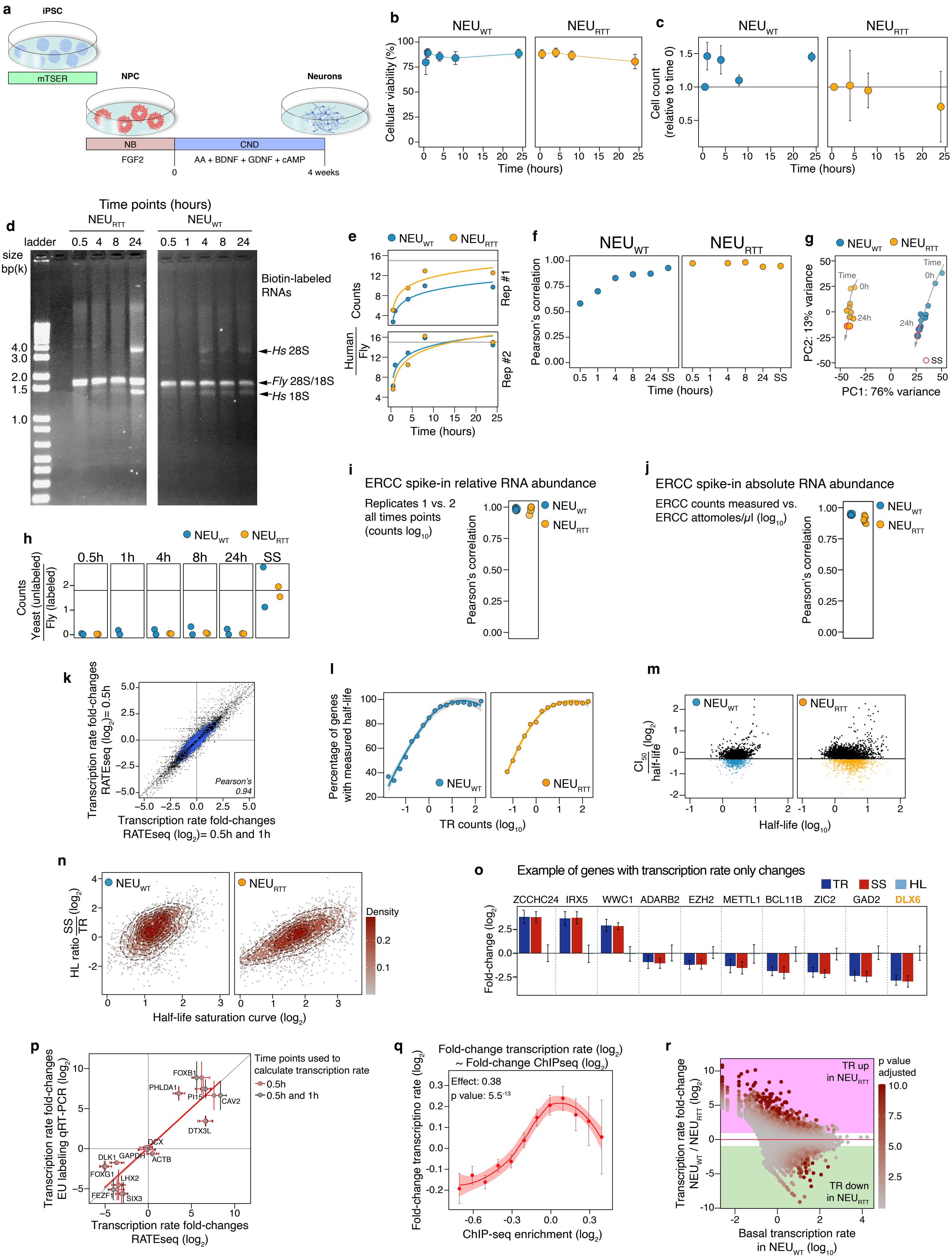
a
iPSC
mTSER
NPC
Neurons
NB
CND
FGF2
AA + BDNF + GDNF + cAMP
0
4 weeks
b
Cellular viability (%)
Time (hours)
c
Cell count (relative to time 0)
Time (hours)
d
Time points (hours)
ladder
size bp(k)
Biotin-labeled RNAs
Hs 28S
Fly 28S/18S
Hs 18S
e
Counts Human/Fly
Rep #1
Rep #2
Time (hours)
f
Pearson's correlation
Time (hours)
g
PC2: 13% variance
PC1: 76% variance
Time
0h
24h
SS
h
Counts Yeast (unlabeled)/Fly (labeled)
0.5h
1h
4h
8h
24h
SS
i
ERCC spike-in relative RNA abundance
Replicates 1 vs. 2 all times points (counts log10)
Pearson's correlation
j
ERCC spike-in absolute RNA abundance
ERCC counts measured vs. ERCC attomoles/µl (log10)
Pearson's correlation
k
Transcription rate fold-changes RATEseq (log2)= 0.5h
Transcription rate fold-changes RATEseq (log2)= 0.5h and 1h
Pearson's 0.94
l
Percentage of genes with measured half-life
TR counts (log10)
m
CI50 (log2) / half-life
Half-life (log10)
n
HL ratio SS/TR (log2)
Half-life saturation curve (log2)
Density
o
Example of genes with transcription rate only changes
TR
SS
HL
ZCCHC24
IRX5
WWC1
ADARB2
EZH2
METTL1
BCL11B
ZIC2
GAD2
DLX6
Fold-change (log2)
p
Transcription rate fold-changes EU labeling qRT-PCR (log2)
Transcription rate fold-changes RATEseq (log2)
Time points used to calculate transcription rate
0.5h
0.5h and 1h
FOXB1
PHLDA1
PI15
CAV2
DTX3L
DCX
GAPDH
ACTB
DLK1
FOXG1
LHX2
FEZF1
SIX3
q
Fold-change transcription rate (log2) ~ Fold-change ChIPseq (log2)
Effect: 0.38
p value: 5.5-13
Fold-change transcriptino rate (log2)
ChIP-seq enrichment (log2)
r
Transcription rate fold-change NEUWT / NEURTT (log2)
TR up in NEURTT
TR down in NEURTT
p value adjusted
Basal transcription rate in NEUWT (log10)

Fig S1. Quality control of the RATE-seq experiment.

A, schematics of the cortical neuronal differentiation protocol including a neuronal enrichment step. B-C, 4sU does not impact neuronal viability (2 independent biological replicates). Error bars= standard deviation. D, representative agarose gel of 4sU-labeled pulled-down RNAs. We run independent gels for each biological replicate (n=2). E, 4sU incorporation kinetics of human mRNA normalized to fly spike-in RNA. F, Pearson's correlation between replicates and time-points of the human RNAs. G, principal component analysis of NEU_{WT} and NEU_{RTT} data showing significant separation between genotypes and time-points. Steady-state samples= red outline and cluster with the 24-hour time-point samples. H, spike-in RNA read counts: unlabeled yeast vs. fly RNA for all time-points. Unlabeled yeast RNA spike-in RNA was used as pull-down negative control. The absence of yeast RNA indicates that the pull-down of 4sU labeled RNAs had minimal contamination of unlabeled human RNAs, but readily detectable in the steady-state samples. I-J, Pearson's correlation of ERCC spike-in RNAs between replicates and spike-in concentration. ERCC spike-in RNA was used as a control for library quality. High Pearson's correlations indicate the quality of the library samples. K, transcription rates measured using only 0.5-hour or 0.5 and 1-hour time-points were highly correlated. L-N, half-life measured with 4sU saturation curve method. (L) percentage of genes with measured half-life depending on transcription rate. Error bars= 95% CI, loess fit. M, the accuracy of half-life shown against half-life magnitude. CI_{50}= 50% confidence interval. Black points denote genes with poorly fit saturation curves and removed from analysis in panel N. N, comparison of half-life estimated with two methods for well-measured genes. O, examples of transcription-mediated changes in steady-state (log2 fold-change and SD bars output from DESeq2). DLX6= high autism-risk gene. P, transcription rate fold-changes measured using 0.5-hour or 0.5- and 1-hour time-points orthogonally validated by 5EU-labeling method. Q, genes with the highest transcription rate fold-change are enriched for MECP2-binding[9]. R, genes with the highest transcription rate increase in NEU_{RTT} had the lowest basal transcription rate in NEU_{WT} neurons[17]. TR= transcription rate, HL= half-life, SS= steady-state. Transcription rates derived from 4 (O and Q) or 2 (P) biological replicates. Error bars= 95% CI. Panel A was created with BioRender.com.

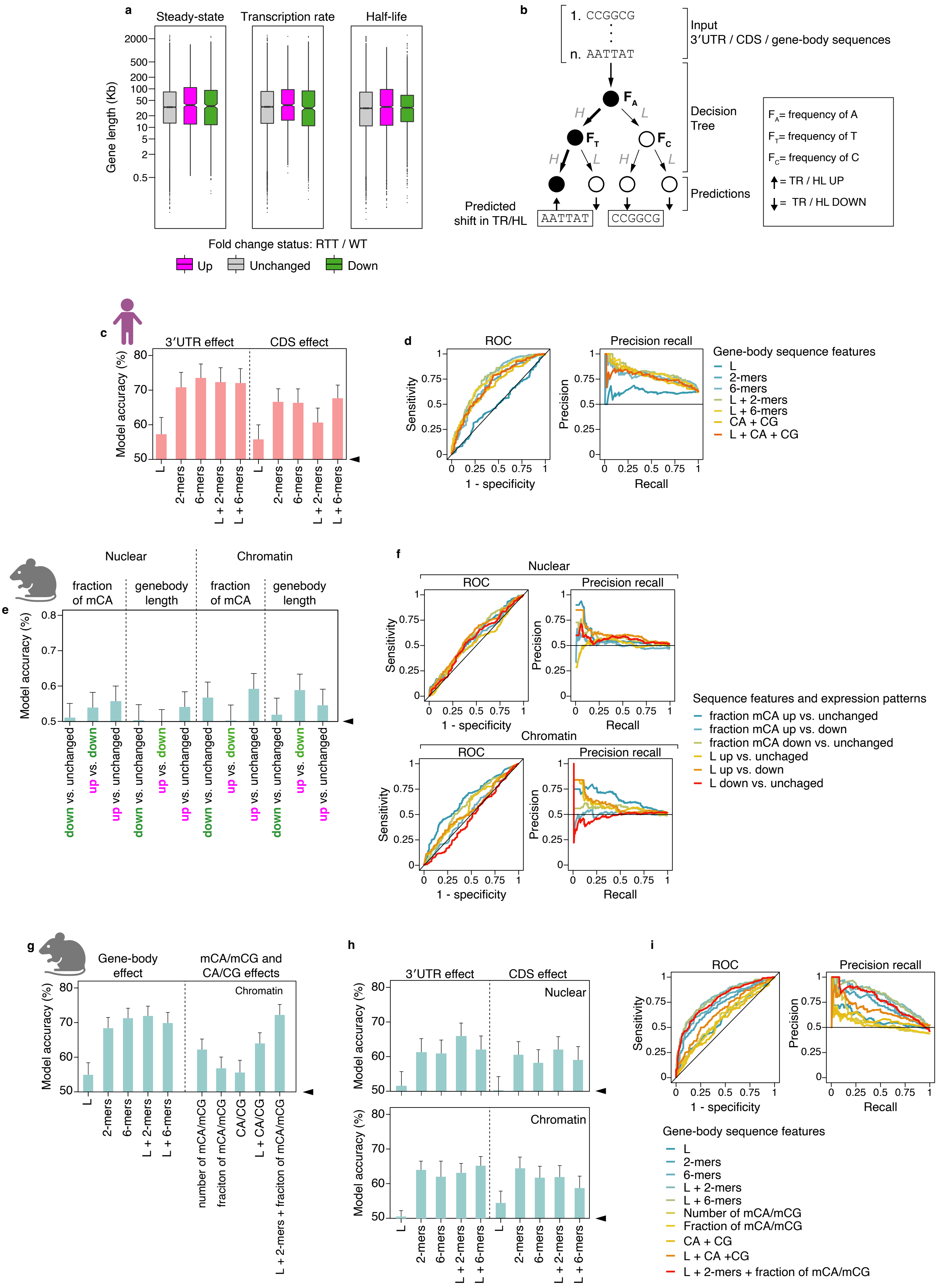
a
Steady-state
Transcription rate
Half-life
Gene length (Kb)
Fold change status: RTT / WT
Up
Unchanged
Down
b
1. CCGGCG
n. AATTAT
Input
3′UTR / CDS / gene-body sequences
Decision Tree
Predictions
Predicted shift in TR/HL
AATTAT
CCGGCG
F_A= frequency of A
F_T= frequency of T
F_C= frequency of C
= TR / HL UP
= TR / HL DOWN
c
3′UTR effect
CDS effect
Model accuracy (%)
L
2-mers
6-mers
L + 2-mers
L + 6-mers
d
ROC
Precision recall
Sensitivity
1 - specificity
Precision
Recall
Gene-body sequence features
L
2-mers
6-mers
L + 2-mers
L + 6-mers
CA + CG
L + CA + CG
e
Nuclear
Chromatin
fraction of mCA
genebody length
down vs. unchanged
up vs. down
up vs. unchanged
f
Nuclear
Chromatin
Sequence features and expression patterns
fraction mCA up vs. unchanged
fraction mCA up vs. down
fraction mCA down vs. unchanged
L up vs. unchaged
L up vs. down
L down vs. unchaged
g
Gene-body effect
mCA/mCG and CA/CG effects
Chromatin
number of mCA/mCG
fraciton of mCA/mCG
CA/CG
L + CA/CG
L + 2-mers + fraciton of mCA/mCG
h
3′UTR effect
CDS effect
Nuclear
Chromatin
i
ROC
Precision recall
Gene-body sequence features
L
2-mers
6-mers
L + 2-mers
L + 6-mers
Number of mCA/mCG
Fraction of mCA/mCG
CA + CG
L + CA +CG
L + 2-mers + fraction of mCA/mCG

Figure S2. Classifier model to predict DNA sequence features affecting transcription rate in the absence of *MECP2*.

A, gene length is not associated with changes in steady-state, transcription rate, or half-life in NEU_{RTT} as seen for adult mice[9] (steady-state and half-life= 2 biological replicates, transcription rate= 4 biological replicates). Up and low hinges= 25th and 75th percentiles. Up and low whiskers= 1.5 *IQR (inter-quartile range) above and below the corresponding hinges. Notches are 1.58 * IQR/sqrt(n), which matches 95% CI for median comparison. B, schematic representation of the classifier decision tree utilized. A collection of decision trees forms the random forest model. C, 3′UTR or CDS mRNA sequence features-based random forest classifier for prediction of transcription rate fold-change in NEU_{RTT}. D, receiver operating characteristic curve (ROC) and precision-recall curve (PRC) showing overall performance of the classifier for prediction of human transcription rate fold-changes. E, predictive model for transcription rate fold-changes in the mice using either nuclear or chromatin-associated RNA samples. While the fraction of mCA and gene-body length offer some accuracy to distinguish transcription rate up versus unchanged or up versus down in some cases, the classifier found that other sequence features offer higher prediction accuracies (see also figure 2C). Error bars= 95% CI. F, ROC and PRC showing overall performance of the classifier for prediction of mouse transcription rate fold-change relative to panel E. G, gene-body or number and frequencies of mCA/mCG mRNA sequence features-based random forest classifier for prediction of transcription rate fold-changes in the mouse *Mecp2 y/-*. Error bars= 95% CI. H, 3′UTR or CDS mRNA sequence features-based random forest classifier for prediction of transcription rate fold-changes in the mouse RTT model. Error bars= 95% CI. I, ROC and PRC show the overall performance of the classifier for predicting mouse transcription rate fold-change based on the nuclear dataset. TR= transcription rate, HL= half-life, SS= steady-state. For panels C, E, G, and H, error bars= 95% CI, and are estimated from approximately 400 genes for human and 500 for mice. Log2 fold-change for each gene is derived from 4 independent replicates (30 minutes and 1h) for human and 10 replicates for mice. Panels C, E, and G were created with BioRender.com.

a

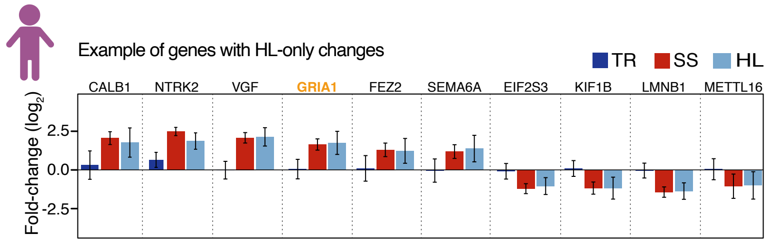

b

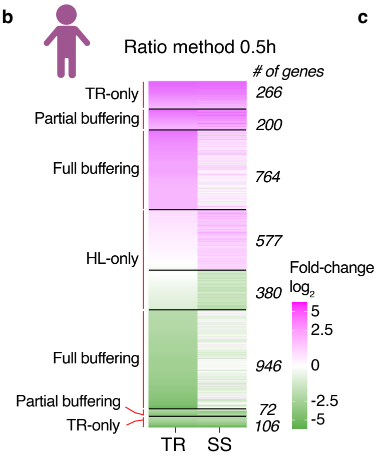

c

Class	# 30 min	# 30 min + 1h	# Overlap	Percentage overlap	# 30 min unique	# 30 min + 1h unique	# Union	# Total	Fisher p value
TR-only (TR up)	266	299	190	71	76	109	375	15,000	3.3^{-290}
Partial buffering (TR up)	200	211	106	53	94	105	305	15,000	2.10^{-152}
Full buffering (TR up)	764	434	146	34	618	288	1,052	15,000	5.50^{-82}
HL-only (SS up)	577	533	461	86	116	72	649	15,000	0.0^{-00}
HL-only (SS down)	380	326	294	90	86	32	412	15,000	0.0^{-00}
Full buffering (TR down)	946	355	127	36	819	288	1,174	15,000	3.40^{-63}
Partial buffering (TR down)	72	87	43	60	29	44	116	15,000	2.40^{-82}
TR only (TR down)	106	145	82	77	24	63	169	15,000	6.50^{-155}
**All buffered genes**	1982	1087	422	39	1,560	665	2,647	15,000	9.2^{-110}

d

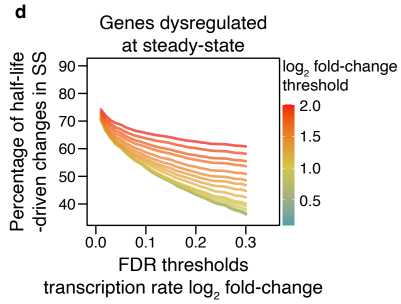

e

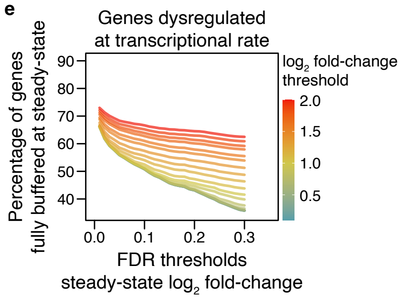

f

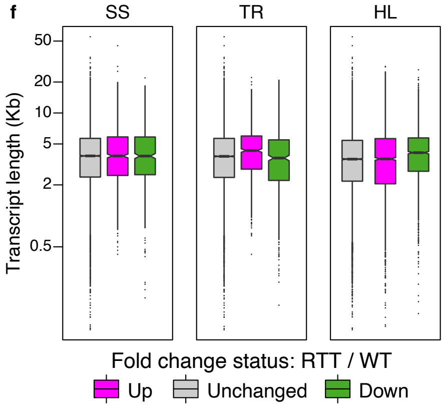

g

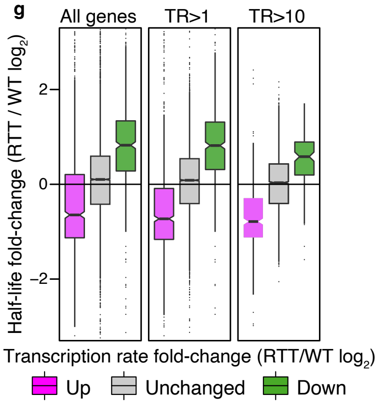

Saturation method

h **i**

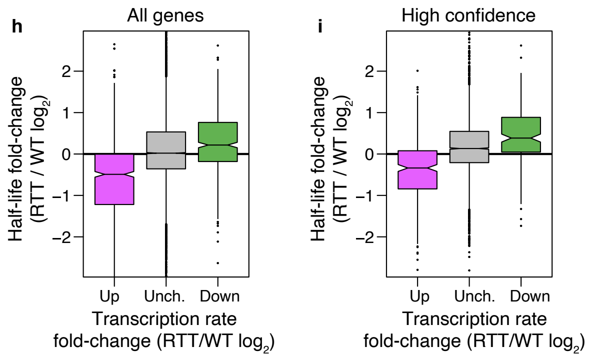

Figure S3. Human RTT neurons show half-life changes in steady-state mRNA levels.

A, DESeq2 outputs (log2) of genes with mRNA half-life only changes, causing changes in the steady-state independent of transcription rate (derived from 2 biological replicates). GRIA1 is known to confer a high risk for autism when mutated. Standard error bars are also output from DESeq2. B, number of genes with changes in transcription rate only (TR-only), partially or fully buffered by half-life changes, and genes whose change in steady-state are caused by altered half-life only (HL-only). These calculations were made using the 0.5h time-point for both cell types, and the figure only shows genes whose transcription rates were altered by at least 4-fold. C, percentage of genes with no significant transcription rate shift or HL-only changes in steady-state (y-axis) as a function of the FDR (x-axis) and fold-change (color) thresholds. D, percentage of genes with no significant steady-state shift or fully buffered by mRNA stability mechanisms (y-axis) as a function of the FDR (x-axis) and fold-change (color) thresholds. E, human genes with steady-state, transcription rate, or half-life fold-changes in NEU_{RTT} do not show significant enrichment for transcript length as seen for adult mice[9]. F, the buffering of transcription rate is observed independently of the underlying abundance levels of measured transcripts. This indicates that most genes transcriptionally dysregulated are buffered by half-life changes independent of their abundances. G-H, the buffering effect is also present when half-life was calculated using the saturation method including all genes (H, low and high standard error bars) or just genes with low standard error bars (I, high confidence). 2 biological replicates for steady-state and half-life, and 4 biological replicates for transcription rate measurements. The transcriptional level changes for each gene are derived from data represented in Figure 3H-I. For F-I, up and low hinges are 25th and 75th percentiles. Up and low whiskers are 1.5 * IQR (inter-quartile range) above and below the corresponding hinges. Notches are 1.58 * IQR / sqrt(n) which matches 95% CI for median comparison. Panels A and B were created with BioRender.com.

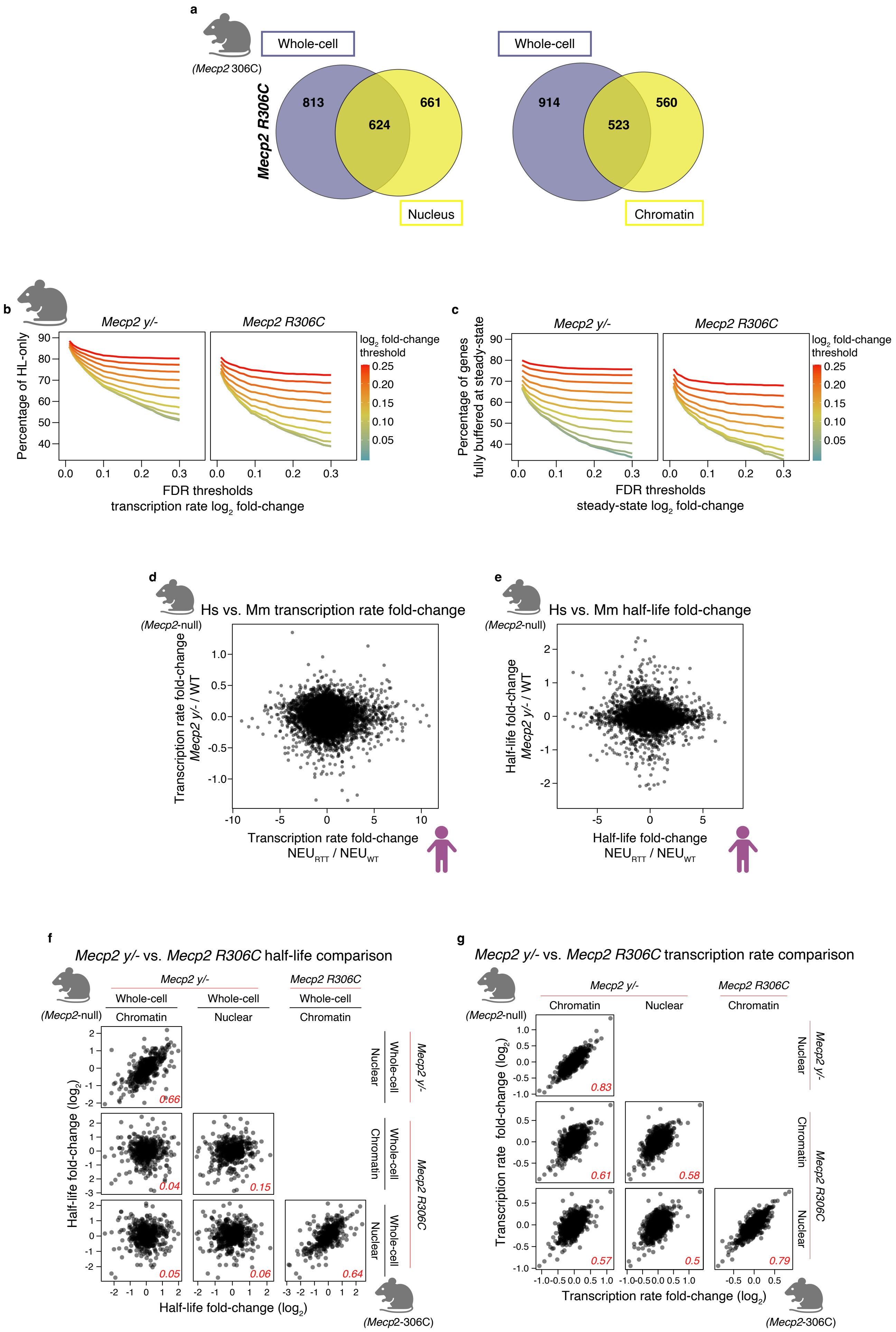

a
(Mecp2 306C)
Mecp2 R306C
Whole-cell
Nucleus
813
624
661
Whole-cell
Chromatin
914
523
560
b
Mecp2 y/-
Mecp2 R306C
Percentage of HL-only
FDR thresholds
transcription rate log2 fold-change
log2 fold-change threshold
0.25
0.20
0.15
0.10
0.05
c
Mecp2 y/-
Mecp2 R306C
Percentage of genes fully buffered at steady-state
FDR thresholds
steady-state log2 fold-change
log2 fold-change threshold
d
(Mecp2-null)
Hs vs. Mm transcription rate fold-change
Transcription rate fold-change Mecp2 y/- / WT
Transcription rate fold-change NEURTT / NEUWT
e
(Mecp2-null)
Hs vs. Mm half-life fold-change
Half-life fold-change Mecp2 y/- / WT
Half-life fold-change NEURTT / NEUWT
f
Mecp2 y/- vs. Mecp2 R306C half-life comparison
(Mecp2-null)
Mecp2 y/-
Mecp2 R306C
Whole-cell
Chromatin
Nuclear
Half-life fold-change (log2)
0.66
0.04
0.15
0.05
0.06
0.64
(Mecp2-306C)
g
Mecp2 y/- vs. Mecp2 R306C transcription rate comparison
(Mecp2-null)
Mecp2 y/-
Mecp2 R306C
Chromatin
Nuclear
Transcription rate fold-change (log2)
0.83
0.61
0.58
0.57
0.5
0.79
(Mecp2-306C)

Figure S4. Mouse RTT models corroborate the human data and show half-life changes in steady-state mRNA levels.

A, the overlap of genes altered at transcription rate based on nuclear (left panel) or chromatin-associated RNA (right panel) and steady-state (whole-cell) in the *Mecp2 R306C* mouse neurons. B, percentage of genes with no significant steady-state shift or fully buffered by half-life (y-axis) as a function of the FDR (x-axis) and fold-change (color) thresholds in the *Mecp2* y/- and *Mecp2 R306C* mice. C, percentage of genes with no significant transcription rate shift or with half-life change (y-axis) as a function of the FDR (x-axis) and fold-change (color) thresholds in the *Mecp2* y/- and *Mecp2 R306C* mice. D-E comparison of the fold-changes between genes in human (Hs) and mouse (Mm) RTT models showing limited agreement in the identity of genes with altered transcription rate (D) and half-life (E) between species. F-G, the identity of genes differentially regulated at half-life and transcription rate is also limited when comparing the *Mecp2* y/- and *Mecp2 R306C* mouse models. Pearson correlations for each comparison are depicted inside boxes in red fonts. TR= transcription rate, HL= half-life, SS= steady-state. Panels A to G were created with BioRender.com.

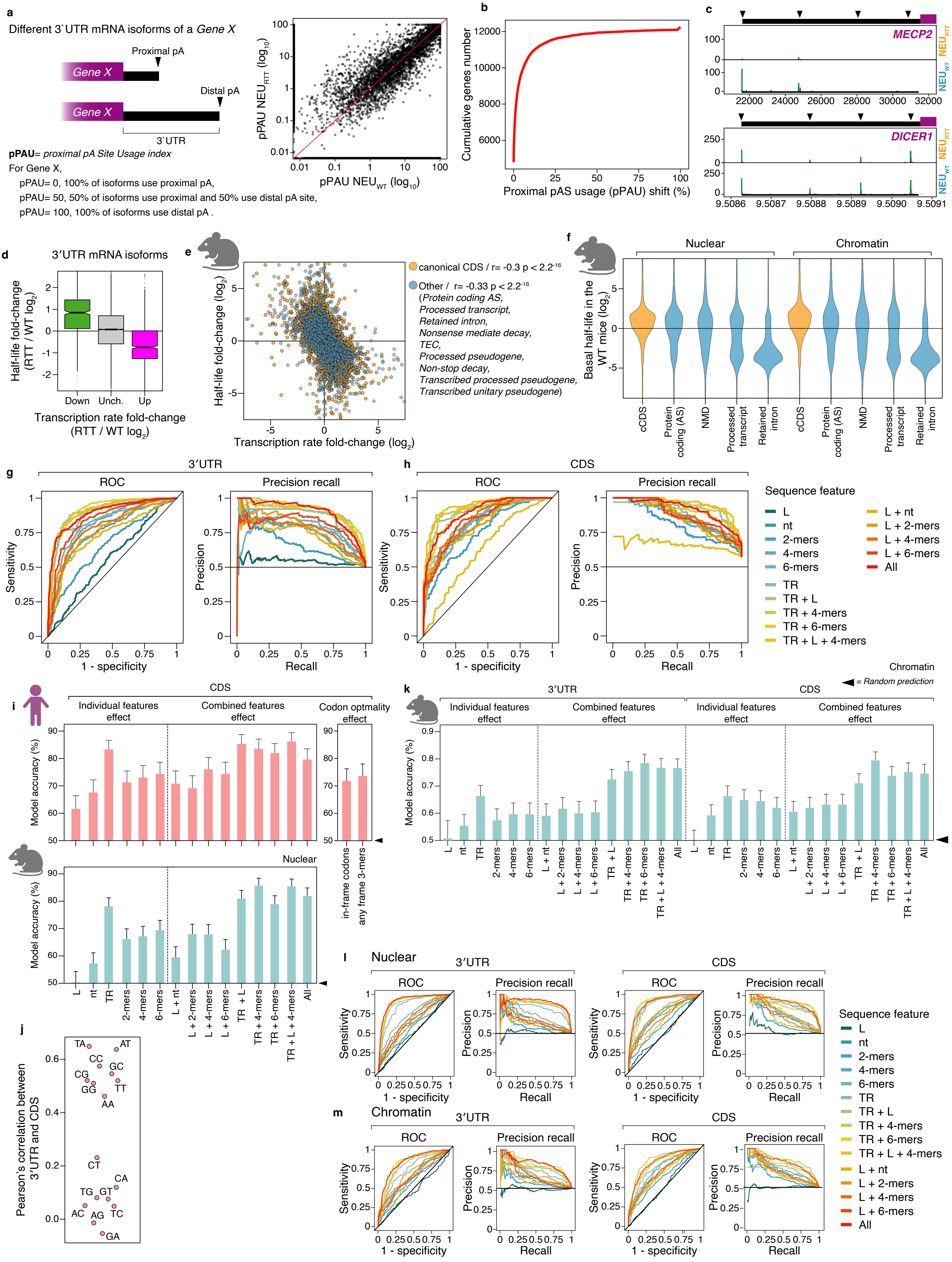

a
Different 3`UTR mRNA isoforms of a Gene X
Proximal pA
Gene X
Distal pA
Gene X
3`UTR
pPAU= proximal pA Site Usage index
For Gene X,
pPAU= 0, 100% of isoforms use proximal pA,
pPAU= 50, 50% of isoforms use proximal and 50% use distal pA site,
pPAU= 100, 100% of isoforms use distal pA .
pPAU NEU_RTT (log10)
pPAU NEU_WT (log10)
b
Cumulative genes number
Proximal pAS usage (pPAU) shift (%)
c
MECP2
DICER1
NEU_RTT
NEU_WT
d
3′UTR mRNA isoforms
Half-life fold-change (RTT / WT log2)
Down
Unch.
Up
Transcription rate fold-change (RTT / WT log2)
e
canonical CDS / r= -0.3 p < 2.2^-16
Other / r= -0.33 p < 2.2^-16
(Protein coding AS,
Processed transcript,
Retained intron,
Nonsense mediate decay,
TEC,
Processed pseudogene,
Non-stop decay,
Transcribed processed pseudogene,
Transcribed unitary pseudogene)
Half-life fold-change (log2)
Transcription rate fold-change (log2)
f
Nuclear
Chromatin
Basal half-life in the WT mice (log2)
cCDS
Protein coding (AS)
NMD
Processed transcript
Retained intron
g
3′UTR
h
CDS
ROC
Precision recall
Sensitivity
1 - specificity
Precision
Recall
Sequence feature
L
nt
2-mers
4-mers
6-mers
TR
TR + L
TR + 4-mers
TR + 6-mers
TR + L + 4-mers
L + nt
L + 2-mers
L + 4-mers
L + 6-mers
All
Chromatin
= Random prediction
i
CDS
Individual features effect
Combined features effect
Codon optmality effect
Model accuracy (%)
in-frame codons
any frame 3-mers
Nuclear
k
3′UTR
CDS
j
Pearson's correlation between 3′UTR and CDS
l
Nuclear
m
Chromatin

Figure S5. Quantification of 3′UTR and alternatively-spliced isoforms in humans and mice, and predictive models of half-life changes in mice.

A, Proximal poly-Adenylation site usage index (pPAU) is the percentage of mRNA isoforms cleaved and poly-Adenylated at the proximal pA site. 0 or 100= all mRNA isoforms of a gene use the proximal site or the distal pA site, respectively. High correlation between pPAU values in NEUWT and NEURTT. B, most genes show small changes <10% in pPAU in NEURTT. C, sequencing read peaks showing the absence of *MECP2* 3′UTR reads in the NEURTT sample and peaks corresponding to *DICER1* pA sites (arrowheads) as an example of genes with similar pPAU index values in NEUWT and NEURTT. D, 3′UTR isoforms also undergo buffering. Data derived from Figures 3H-I (10 biological replicates). Up and low hinges= 25th and 75th percentiles. Up and low whiskers= 1.5*IQR above and below the hinges. Notches= 1.58*IQR/sqrt(n), matching 95% CI for median comparison. E, Pearson's correlation of alternatively-spliced mRNAs in the *Mecp2* y/- mouse shows that canonical CDS and non-protein-coding isoforms undergo buffering. F, quantification of mRNA isoforms stability in WT mice using nuclear or chromatin-associated RNA confirms that nonsense-mediated decay, processed transcripts, and retained-intron mRNAs are less stable than the canonical CDS and alternatively-spliced protein-coding isoforms. This underscores the quality of the mRNA stability calculations based on whole-cell versus nuclear or chromatin-associated mRNA ratios. G-H, ROC and PRC for predicting stability changes based on 3′UTR (G) or CDS (H). I, CDS-specific features random forest predicting stability changes for human and *Mecp2* y/- mouse. Error bars= 95% CI. J, Pearson's correlation shows significant similarities between sequence composition between CDS and 3′UTRs. K, 3′UTR- features random forest predicting stability changes for *Mecp2* y/- mouse based on chromatin-associated RNAs. Error bars= 95% CI. L, sequence features random forest predicting stability changes based on chromatin-associated RNAs in *Mecp2* y/- mouse model, indicating that 3′UTR and CDS contain highly accurate predicting elements for stability changes. Error bars= 95% CI. M-N, ROC and PRC classifier performance for half-life fold-change prediction in *Mecp2* y/- mouse based on nuclear (M) or chromatin-associated RNA (N). TR= transcription rate, HL= half-life, SS= steady-state. Steady-state and half-life= 2 biological replicates; transcription rate= 4 biological replicates. Panels E, F, I, and K were created with BioRender.com.

Primer list for qRT-PCRs

CAV2	Forward	ATTCTCTTTGCCACCCTCAG
	Reverse	GTCCTACGCTCGTACACAATG
PI15	Forward	TCGCAGAATGACATGATCGC
	Reverse	TGGTCCCAAATGCAAGTAGC
FOXB1	Forward	CGCGCAACTTGAAGCAAC
	Reverse	TCAGCGAGATGTACGAGTAGG
DTX3L	Forward	AAAGGAAATCAGCCAGAGGG
	Reverse	GGGTATCTCTTTCCTGGGTTTG
PHLDA1	Forward	ACCAAATACCGCACCCAC
	Reverse	AGAAATGTGCTCGTCCCAC
FOXG1	Forward	CCTGCCCTGTGAGTCTTTAAG
	Reverse	GTTCACTTACAGTCTGGTCCC
LHX2	Forward	GGTCTTCCCTACTACAATGGC
	Reverse	GTCGTTTTCGTTGCAGCTTAG
FEZF1	Forward	CTGTGGCAAAGGGTTTCATC
	Reverse	TCTTGTCGTTGTGGGTGTG
SIX3	Forward	CAACCCCAGCAAGAAACG
	Reverse	CTCGGTCCAATGGCCTG
DLK1	Forward	CGAGGATGACAATGTTTGCAG
	Reverse	CAGAGTCCGTGAAGGCAG
DCX	Forward	TATGCGCCGAAGCAAGTCTCCA
	Reverse	CATCCAAGGACAGAGGCAGGTA
ACTB	Forward	TGAAGTGTGACGTGGACATC
	Reverse	GGAGGAGCAATGATCTTGAT
GAPDH	Forward	CATGAGAAGTATGACAACAGCCT
	Reverse	AGTCCTTCCACGATACCAAAGT
AGO3	Forward	TCCTGTTGGGAGGCAAATAACA
	Reverse	AAGAGTAGTGGTTCTGTCACAGA
FZD3	Forward	GCTTTGCACTCTGCTCTTGTAG
	Reverse	TTGTACACTCACAGTTAAAGTGCT
NT5DC2	Forward	CAACCCCACCTACTTCTCAAGG
	Reverse	GTAGAAGGTGAAGTCCACGCG
KIF3A	Forward	TGAGTAATCAAGGGAAGGGTCG
	Reverse	AAAACAACTCCCTTTCTCCAGA
RBL2	Forward	AAACTTATGACCTCTTCCTTTAGG
	Reverse	TTTTAAACTGCCAGGAACACCC
SKP2	Forward	GTTGCACAGGAAATGATGATGCT
	Reverse	AACCCCAGCTCTTGTCACTAAT
18S	Forward	GATGGGCGGCGGAAAATAG
	Reverse	GCGTGGATTCTGCATAATGGT
