# Supplementary material for: Buffering of transcription rate by mRNA half-life is a conserved feature of Rett syndrome models
Source: Nat Commun. 2023 Apr 5;14:1896. doi: 10.1038/s41467-023-37339-6 (PMC10076348; doi:10.1038/s41467-023-37339-6)
Supplement: Supplementary file 3 — Description of Additional Supplementary Files [file 41467_2023_37339_MOESM3_ESM.pdf]

Description of Additional Supplementary Files

File Name: Supplementary Data 1
Description: log2 fold-change in transcription rates at gene and mRNA isoform levels

File Name: Supplementary Data 2
Description: log2 fold-change in half-life at gene and mRNA isoform levels

File Name: Supplementary Data 3
Description: List of studies that measured mRNA half-life in different cell types and organisms.

File Name: Supplementary Data 4
Description: List of gene names generated from half-life quantification method using either 0.5h or 0.5 and 1h time points.

File Name: Supplementary Data 5
Description: log2 fold-change in mRNA stability at gene and mRNA isoform levels for Rett syndrome mice models quantified from datasets published by Boxer et al.

File Name: Supplementary Data 6
Description: log2 fold-change in steady-state and transcriptional rates of miRNAs in human Rett syndrome neurons.

File Name: Supplementary Data 7
Description: Enrichment scores of miRNAs and RNA-binding protein binding sites found for mRNAs undergoing buffering.
