# Supplementary material for: Buffering of transcription rate by mRNA half-life is a conserved feature of Rett syndrome models
Source: Nat Commun. 2023 Apr 5;14:1896. doi: 10.1038/s41467-023-37339-6 (PMC10076348; doi:10.1038/s41467-023-37339-6)
Supplement: Supplementary file 11 — Reporting Summary [file 41467_2023_37339_MOESM11_ESM.pdf]

nature portfolio

Corresponding author(s): James Ellis

Last updated by author(s): Jan 30, 2023

Reporting Summary

Nature Portfolio wishes to improve the reproducibility of the work that we publish. This form provides structure for consistency and transparency in reporting. For further information on Nature Portfolio policies, see our Editorial Policies and the Editorial Policy Checklist.

Statistics

For all statistical analyses, confirm that the following items are present in the figure legend, table legend, main text, or Methods section.

n/a	Confirmed	
☐	☒	The exact sample size (*n*) for each experimental group/condition, given as a discrete number and unit of measurement
☐	☒	A statement on whether measurements were taken from distinct samples or whether the same sample was measured repeatedly
☐	☒	The statistical test(s) used AND whether they are one- or two-sided<br>*Only common tests should be described solely by name; describe more complex techniques in the Methods section.*
☐	☒	A description of all covariates tested
☐	☒	A description of any assumptions or corrections, such as tests of normality and adjustment for multiple comparisons
☐	☒	A full description of the statistical parameters including central tendency (e.g. means) or other basic estimates (e.g. regression coefficient) AND variation (e.g. standard deviation) or associated estimates of uncertainty (e.g. confidence intervals)
☐	☒	For null hypothesis testing, the test statistic (e.g. *F*, *t*, *r*) with confidence intervals, effect sizes, degrees of freedom and *P* value noted<br>*Give P values as exact values whenever suitable.*
☒	☐	For Bayesian analysis, information on the choice of priors and Markov chain Monte Carlo settings
☐	☒	For hierarchical and complex designs, identification of the appropriate level for tests and full reporting of outcomes
☒	☐	Estimates of effect sizes (e.g. Cohen's *d*, Pearson's *r*), indicating how they were calculated

Our web collection on statistics for biologists contains articles on many of the points above.

Software and code

Policy information about availability of computer code

Data collection	stats v4.0.5<br>cutadapt v1.10<br>STAR v2.6.0c<br>liftOver (UCSC)<br>pybedtools v0.9.0<br>pysam v0.19.0<br>pypiper v0.12.2<br>Rsubread v2.2.6<br>mirdeep2 v0.1.3<br>DESeq2 v1.30.0<br>preprocessCore v1.50.0<br>Biostrings v2.56.0<br>randomForest v4.6.14<br>precrec v.0.12.7<br>transite v1.16.0<br>ImageStudio v5.2.5
Data analysis	We state and cite all packages and software tools in the Methods section. Software was obtained from publicly available sources; papers describing the software are cited in the methods section. Computer code used is available at https://github.com/JellisLab/stabilome-rett

For manuscripts utilizing custom algorithms or software that are central to the research but not yet described in published literature, software must be made available to editors and reviewers. We strongly encourage code deposition in a community repository (e.g. GitHub). See the Nature Portfolio guidelines for submitting code & software for further information.

Data

Policy information about availability of data

All manuscripts must include a data availability statement. This statement should provide the following information, where applicable:

- Accession codes, unique identifiers, or web links for publicly available datasets
- A description of any restrictions on data availability
- For clinical datasets or third party data, please ensure that the statement adheres to our policy

The original sequencing data that support the findings of this study can be accessed from GEO using the access number GSE191168 (https://www.ncbi.nlm.nih.gov/geo/query/acc.cgi?acc=GSE191168). The mouse data reanalyzed in this study can be downloaded from GSE128178 (https://www.ncbi.nlm.nih.gov/geo/query/acc.cgi?acc=GSE128178) (whole-cell, nuclear and chromatin RNA-seq) and GSE139509 (https://www.ncbi.nlm.nih.gov/geo/query/acc.cgi?acc=GSE139509 (MECP2 ChIP-seq). Source data are provided with this paper.

Other databases utilized in this work:
gencode v29 (https://www.gencodegenes.org/human/)
all-r6.22 (https://flybase.org/)
saccharomyces_cerevisiae.gff (https://yeastgenome.org/)
hg38 (https://hgdownload.soe.ucsc.edu/downloads.html)
dm6 (https://flybase.org/)
sacCer3 (https://hgdownload.soe.ucsc.edu/goldenPath/sacCer3/bigZips/)
ERCC (https://www.thermofisher.com/order/catalog/product/4456740)
PolyA_DB v3 (https://exon.apps.wistar.org/PolyA_DB/v3/)
Pri-miRNA annotation (https://genome.cshlp.org/content/25/9/1401/suppl/DC1)

Field-specific reporting

Please select the one below that is the best fit for your research. If you are not sure, read the appropriate sections before making your selection.

☒ Life sciences ☐ Behavioural & social sciences ☐ Ecological, evolutionary & environmental sciences

For a reference copy of the document with all sections, see nature.com/documents/nr-reporting-summary-flat.pdf

Life sciences study design

All studies must disclose on these points even when the disclosure is negative.

Sample size	Sample size for each experiment is indicated in the figure legends and methods. No sample-size calculations were performed in our experiments. Sample size was determined based on the current minimal requirements in the literature (e.g. for RNA-seq), providing adequate consistency of measured differences between groups.
Data exclusions	No data were excluded from analyses.
Replication	Most experiments were repeated in duplicate with similar results. All experiments were replicated as stated in the legends and methods.
Randomization	No randomization was employed. We don't assign treatments to samples or individuals. Allocation was not utilized in this study.
Blinding	Blinding is not relevant to this study since group allocation does not occur. Data reported for these experiments is not subjective, but based on data acquisition and analysis approaches established in literature.

Reporting for specific materials, systems and methods

We require information from authors about some types of materials, experimental systems and methods used in many studies. Here, indicate whether each material, system or method listed is relevant to your study. If you are not sure if a list item applies to your research, read the appropriate section before selecting a response.

Materials & experimental systems

n/a	Involved in the study
☐	☒ Antibodies
☒	☐ Eukaryotic cell lines
☒	☐ Palaeontology and archaeology
☒	☐ Animals and other organisms
☒	☐ Human research participants
☒	☐ Clinical data
☒	☐ Dual use research of concern

Methods

n/a	Involved in the study
☒	☐ ChIP-seq
☒	☐ Flow cytometry
☒	☐ MRI-based neuroimaging

Antibodies

Antibodies used

Biotin Mouse Anti-Human CD44, BD Biosciences, Clone G44-26; Cat# 555477.
Biotin Mouse Anti-Human CD184, BD Biosciences, Clone 12G5; Cat# 555973.
Rabbit Anti-MECP2, Millipore, Cat# 07-013; RRID:AB_2144004
Mouse Anti-bActin, Sigma-Aldrich, Cat# A5441; RRID:AB_476744
IRDye 800CW Donkey anti-Mouse IgG, Cat# 926-32212, LI-COR
IRDye 680RD Donkey anti-Rabbit IgG, Cat# 926-68073, LI-COR

Validation

All antibodies used in this study are commercially available and of standard use in the field.
Validation of MECP2 antibody is confirmed by absence of signal in the human Neuron Rett syndrome sample, which is a knock-out for MECP2 gene.
The anti-bActin antibody #A5441 has been extensively validated is human protein samples in the literature (e.g., https://www.citeab.com/antibodies/2304864-a5441-monoclonal-anti-beta-actin-antibody-produced-i).
Mouse Anti-Human CD44 and 184 have been extensively validated is human cell separation in the literature (e.g., https://www.citeab.com/antibodies/2408548-559942-bd-pharmingen-apc-mouse-anti-human-cd44?des=9ee0a5dcc6e651fb; and https://www.citeab.com/antibodies/2410756-555974-bd-pharmingen-pe-mouse-anti-human-cd184?des=c6460f000dd732e2)
